# Additional chemoradiotherapy following endoscopic submucosal dissection in patients with esophageal squamous cell carcinoma: a narrative review

**DOI:** 10.3389/fonc.2025.1527634

**Published:** 2025-06-16

**Authors:** Yan Lin, Shou-Feng Wang, Huan-Wei Liang, Yang Liu, Wei Huang, Xin-Bin Pan

**Affiliations:** ^1^ Department of Gastroenterology, Jiangbin Hospital of Guangxi Zhuang Autonomous Region, Nanning, Guangxi, China; ^2^ Department of Thoracic Surgery, Guangxi Medical University Cancer Hospital, Nanning, Guangxi, China; ^3^ Department of Radiation Oncology, Guangxi Medical University Cancer Hospital, Nanning, Guangxi, China

**Keywords:** esophageal cancer, endoscopic submucosal dissection, ESD, surgery, radiotherapy, chemotherapy

## Abstract

This review offers a critical synthesis of additional therapeutic strategies following endoscopic submucosal dissection (ESD) for esophageal squamous cell carcinoma, providing evidence-based recommendations to optimize clinical decision-making. For pT1a-EP/LPM lesions, ESD alone demonstrates curative potential with lymph node metastasis rates ranging from 0.0% to 3.3%. In contrast, pT1b-MM tumors exhibiting lymphovascular invasion warrant adjuvant chemoradiation therapy, associated with 21.4% nodal metastasis rates. For pT1b-SM1 lesions, chemoradiation is indicated-particularly demonstrating 13.2% nodal involvement without lymphovascular invasion versus 60.0% metastasis risk in cases with vascular invasion during observation. Timing of additional chemoradiotherapy should be expedited, with immediate initiation (1–2 months post-ESD) showing superior outcomes. Radiation dosing optimization reveals equivalent efficacy between ​​lower radiation doses (40-41.4 Gy) and higher doses (50-50.4 Gy), with reduced treatment-related toxicity. Target volume delineation should prioritize the ESD bed with appropriate margins over elective nodal coverage, maintaining therapeutic efficacy while minimizing radiation exposure. The role of concurrent chemotherapy remains controversial, with retrospective evidence suggesting definitive radiotherapy may provide comparable local control.

## 1 Introduction

Esophageal cancer persists as a major global health challenge, ranked seventh among malignancies worldwide with over 470,000 annual diagnoses (1). Histologically classified cases reveal esophageal squamous cell carcinoma predominates in 90% of instances, exhibiting disproportionately high prevalence in East Asia. Notably, China bears half of the global squamous cell carcinoma burden (2). With advancements in screening technologies and early diagnostic modalities, a growing proportion of patients are identified at earlier disease stages (3).

Endoscopic submucosal dissection (ESD) has emerged as a cornerstone intervention for superficial esophageal carcinoma (4). This technique offers distinct clinical advantages, particularly its superior en bloc resection rates and capacity for precise histopathological assessment. By enabling localized tumor excision while concurrently evaluating critical lymph node metastasis risk factors, including invasion depth, lymphovascular invasion, and invasion pattern, ESD is poised to expand its role in minimally invasive esophageal cancer management.

Nevertheless, therapeutic misjudgment arising from inaccurate indication assessment or curability evaluation may lead to treatment failure, necessitating additional therapeutic interventions post-ESD (5). Current evidence remains insufficient regarding the efficacy of additional chemoradiotherapy following ESD, with several pivotal clinical questions requiring resolution. These clinical uncertainties specifically concern: (1) optimal patient selection criteria for additional chemoradiotherapy, (2) timing of additional chemoradiotherapy post-ESD, (3) radiation dose optimization strategies, (4) target volume delineation strategies, and (5) therapeutic value of concurrent chemotherapy.

To addressing these critical knowledge gaps, we conducted this review adhering to the Preferred Reporting Items for Systematic Reviews and Meta-analyses reporting guidelines (6, 7), through comprehensive searches of Embase, PubMed, and Cochrane Library databases from inception through April 2025. Employing predefined search terms (“esophageal cancer”, “endoscopic submucosal dissection”, “radiotherapy”), we critically appraised relevant studies. Our synthesis of current evidence aims to provide evidence-based recommendations for optimizing post-ESD therapeutic protocols and guiding future clinical investigations.

## 2 Diagnostic accuracy

Current guidelines establish clear indications for ESD in esophageal squamous cell carcinoma: (1) clinical epithelial/lamina propria mucosae (T1a-EP/LPM) lesions, (2) circumferential T1a-EP/LPM lesions ≤50 mm, and (3) clinical muscularis mucosae (T1a-MM) or submucosa invasion ≤200 μm (T1b-SM1) (8–10). While image-enhanced magnifying endoscopy and iodine staining reliably assess lateral lesion extension, accurate determination of invasion depth remains a critical challenge.

Standard diagnostic modalities for invasion depth evaluation include endoscopic ultrasound and magnifying endoscopy (10), with their diagnostic performance detailed in **Table 1**. Among lesions classified as cMM/SM1 through magnifying endoscopy (type B2 vessels), pathological staging demonstrated: 27.4% pEP/LPM, 55.7% pMM/SM1, and 17.0% submucosa invasion >200 μm (pSM2) (11–14). For those diagnosed via type V3 vessel patterns, corresponding pathological distributions were 29.8% pEP/LPM, 42.3% pMM/SM1, and 27.9% pSM2 (15). Notably, the corresponding pathological diagnoses were: 55.2% pEP/LPM, 29.3% pMM/SM1, and 15.5% pSM2 for endoscopic ultrasound-based cMM/SM1. Furthermore, 15.5%-27.9% of cMM/SM1 cases prove to be pSM2.

**Table 1:** Diagnostic accuracy of cancer invasion depth using endoscopy and endoscopic ultrasound.

cMM/SM1	pEP/LPM	pMM/SM1	pSM2
B2 vessels (11–14)	58/212 (27.4%)	118/212 (55.7%)	36/212 (17.0%)
V3 vessels (15)	31/104 (29.8%)	44/104 (42.3%)	29/104 (27.9%)
Endoscopic ultrasound (11)	32/58 (55.2%)	17/58 (29.3%)	9/58 (15.5%)

T1a-EP/LPM: Epithelial/lamina propria mucosae esophageal cancer, T1a-MM: muscularis mucosae esophageal cancer, T1b-SM1: submucosa invasion ≤ 200 μm, T1b-SM2: submucosa invasion > 200 μm.

B1 vessels: characterized by small, irregular, dot-like microvessels without loop formation, indicates tumor invasion confined to the T1a-EP/LPM.

B2 vessels: marked by severe irregularity in microvessel morphology, suggests invasion into the T1a-MM or T1b-SM1.

B3 vessels: characterized by complete destruction of microvessel structures, indicates deeper invasion into T1b-SM2.

V1 vessels: tumor adhesion to or indentation of a vessel without clear evidence of invasion into the vessel wall, suggests non-invasive or suspicious for invasion.

V2 vessels: tumor invasion into the vessel wall, up to the adventitia, indicates local tumor progression and increased risk of metastatic spread.

V3 vessels: tumor invasion through the vessel wall with luminal occlusion or thrombus formation, indicates invasion into T1b-SM1 or deeper.

Vn vessels: ​newly formed tumor vessels, indicates invasion into mid-to-deep submucosa or muscularis propria.

These findings reveal substantial discrepancies between preoperative assessments and postoperative findings. This disparity arises from ​​operator-dependent interpretative variability in assessing deep invasion patterns​​, compounded by ​​inherent limitations in current imaging modalities. Therefore, additional treatments following ESD should be primarily determined by pathological factors, particularly invasion depth and the presence of lymphovascular invasion.

## 3 Additional treatments

Patients with completely resected pEP/LPM carcinomas exhibit minimal lymph node metastasis risk (0.0-3.3%) (8). alone provides curative intent in these cases, with annual endoscopic surveillance recommended for early detection of metachronous lesions rather than additional therapy.

In pMM carcinomas without lymphovascular invasion, (**Table 2**) nodal metastasis rates differ significantly across management strategies: 5.6% (95% confidence interval [CI]: 2.9-9.5%) under observation (16–20), 0.0% (95% CI: 0.0-46.0%) with surgical resection (21–23), and 5.9% (95% CI: 0.2–28.7%) following chemoradiotherapy (24–27).

**Table 2:** Metastasis rates in patients with muscularis mucosae cancers underwent endoscopic submucosal dissection.

Lymphovascular invasion status	Observation (16–20)	Additional surgery (21–23)	Additional chemoradiotherapy (24–27)
Non-lymphovascular invasion	12/216 (5.6%)	0/6 (0.0%)	1/17 (5.9%)
Lymphovascular invasion	3/14 (21.4%)	1/20 (5.0%)	7/45 (15.6%)

CI, confidence interval.

For lymphovascular invasion positive pMM cases, corresponding rates increase to 21.4% (95% CI: 4.7-50.8) (18–20), 5.0% (95% CI: 0.1–24.9%) (21–23), and 15.6% (95% CI: 6.5–29.5%) (24–27). Given surgical mortality risks and chemoradiation-related grade ≥3 toxicities, additional treatments are not recommended in patients without lymphovascular invasion, but essential for lymphovascular invasion positive cases.

Submucosal invasive carcinomas demonstrate distinct metastatic patterns (**Table 3**). For pSM1 lesions without lymphovascular invasion, surveillance yields 13.2% metastasis (16–20), additional surgery shows 0.0% metastasis (21–23), and chemoradiation yields 2.9% metastasis (24–27). In contrast, lymphovascular invasion positive cases show 60.0%, 0.0%, and 17.9%, respectively.

**Table 3:** Metastasis rates in patients with submucosa invasion cancers underwent endoscopic submucosal dissection.

Lymphovascular invasion status	Observation (16–20)	Additional surgery (21–23)	Additional chemoradiotherapy (24–27)
pSM1			
Non-lymphovascular invasion	5/38 (13.2%)	0/5 (0.0%)	1/35 (2.9%)
Lymphovascular invasion	3/5 (60.0%)	0/14 (0.0%)	5/28 (17.9%)
pSM2			
Non-lymphovascular invasion	3/16 (18.8%)	1/12 (8.3%)	8/86 (9.3%)
Lymphovascular invasion	0/4 (0.0%)	0/21 (0.0%)	23/82 (28.1%)

SM1, submucosa invasion ≤ 200 μm; SM2, submucosa invasion > 200 μm.

In pSM2 cohorts, observation achieves 18.8% metastasis without lymphovascular invasion and 0.0% with lymphovascular invasion (16–20, 28). Additional surgery shows 8.3% and 0.0%, respectively (21–23). Additional chemoradiotherapy resulted in metastasis rates of 9.3% and 28.1%, respectively (24–27, 29).

**Figure 1:**
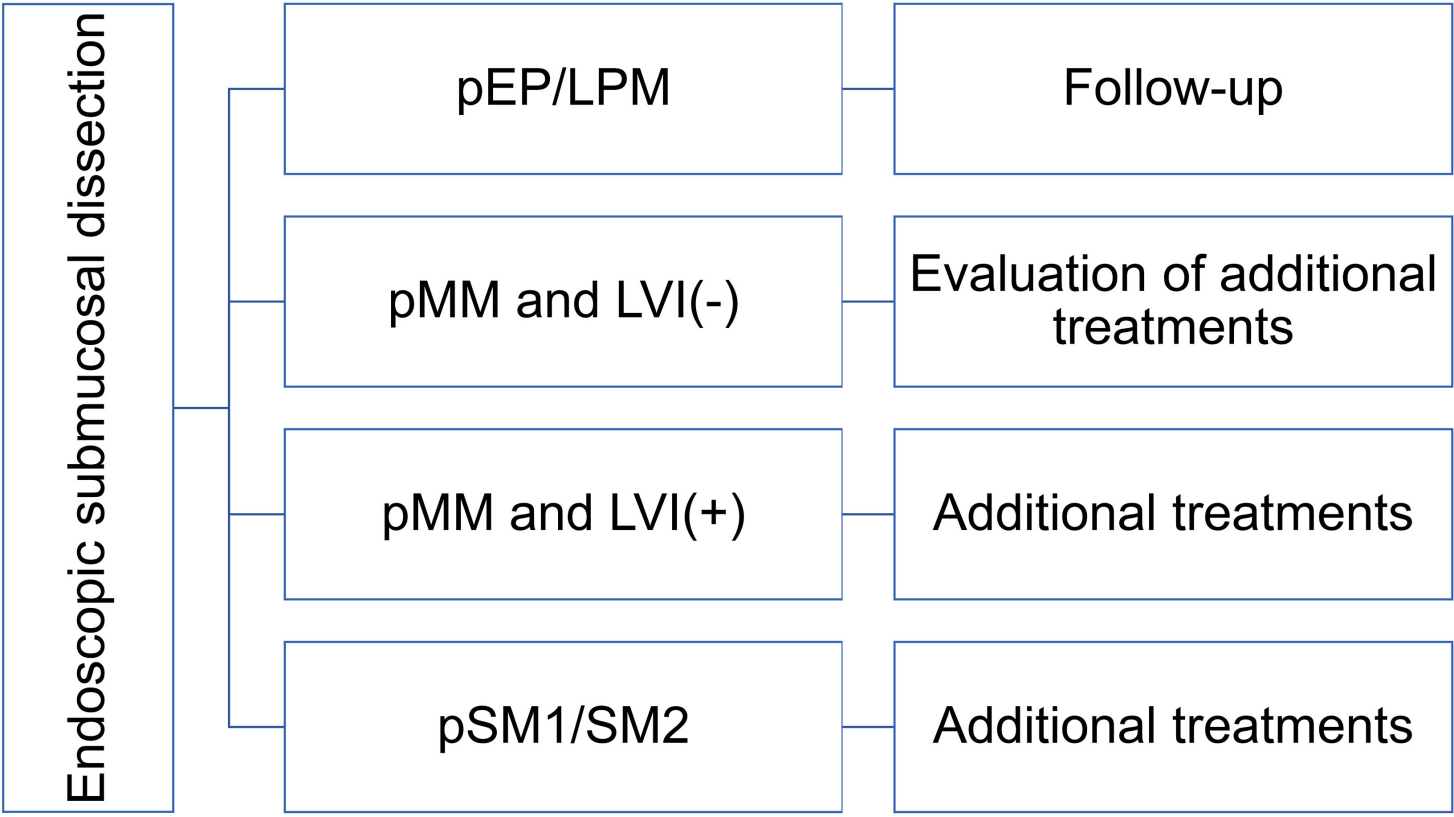
Recommendations of additional treatments after endoscopic submucosal dissection. T1a-EP/LPM: epithelial/lamina propria mucosae esophageal cancer, T1a-MM: muscularis mucosae esophageal cancer, T1b-SM1: submucosa invasion ≤ 200 μm, T1b-SM2: submucosa invasion > 200 μm, LVI: lymphovascular invasion.

The clinically elevated metastasis risk under observation versus additional interventions in lymphovascular invasion negative pSM carcinomas justifies adjuvant treatment despite potential side effects. Similarly, lymphovascular invasion positive subgroups require additional management, notwithstanding paradoxical outcome variations in pSM2 lymphovascular invasion cohorts. Based on these findings, recommendations for additional treatments are outlined in **Figure 1**.

## 4 Additional chemoradiotherapy vs. surgery

Both esophagectomy and chemoradiotherapy serve as primary additional interventions (16, 19). Esophagectomy demonstrates favorable 3-year disease-free survival (86%) (30), 5-year disease-free survival (100%) (31), and 5-year overall survival (90-100%) (22, 31). Compared to upfront esophagectomy, ESD followed by surgery achieves equivalent 3-year overall survival (91.6% vs. 90.9%, hazard ratio [HR] = 0.88, 95% CI: 0.24–3.21; P = 0.871) (32). Furthermore, esophagectomy allows for a more comprehensive assessment of the primary tumor site and regional lymph node status, addressing a limitation of ESD. These outcomes establish esophagectomy as the current therapeutic standard.

Nevertheless, surgical risks remain substantial, with 1.3% treatment-related mortality (95% CI: 0.7-2.2%) (33–37). Additionally, grade 3 and 4 adverse events were reported, including 6.3% anastomotic leaks, 7.7% pneumonia, 2.9% recurrent nerve palsy, and 1.9% fistulae (38). These complications, coupled with quality of life impairment, necessitate cautious patient selection, particularly in elderly or comorbid populations.

Additional chemoradiotherapy emerges an alternative to esophagectomy. The JCOG0508 trial reported a 3-year overall survival rate of 90.7% (90% CI: 84.0-94.7%) for cSM1/SM2 carcinomas managed with ESD plus chemoradiotherapy (39). Real-world evidence from Japanese multicenter studies reveals comparable 5-year overall survival (HR = 0.72, 95% CI: 0.31-1.68; P = 0.44), relapse-free survival (HR = 0.70, 95% CI: 0.34-1.41; P = 0.31), and cause-specific survival (HR = 0.86, 95% CI: 0.08–9.47; P = 0.90) between chemoradiotherapy and esophagectomy (5). Retrospective analyses consistently confirm equivalent survival benefits across both approaches (17, 21–23, 30, 40–43).

Notably, chemoradiotherapy demonstrates superior safety profiles, with grade ≥2 toxicities (dyspnea 11.1%, esophagitis 2.7%, cardiac events 2.7-1.4%) and grade ≥3 stenosis (0.6%) being significantly rarer than surgical complications (39, 44). Moreover, chemoradiotherapy further enhances quality of life metrics versus esophagectomy (45). Salvage surgery post-recurrence maintains comparable efficacy to primary esophagectomy, with 90-day mortality rates of 4% versus 5%.

Current clinical practice reflects these advantages, with 61.5% receiving chemoradiotherapy and 24.7% undergoing esophagectomy (5). The ongoing Ad-ESD randomized trial (NCT04616157) directly comparing chemoradiotherapy versus esophagectomy in cN0-pT1b esophageal squamous cell carcinoma will provide Level I evidence to optimize treatment algorithms (46).

## 5 Key clinical uncertainties of additional chemoradiotherapy

### 5.1 Optimal timing of chemoradiotherapy post-ESD

The optimal timing for initiating adjuvant chemoradiotherapy following ESD remains undefined. Current evidence supports initiating treatment within 1–2 months post-procedure, mirroring esophagectomy adjuvant therapy intervals (39, 47, 48). This empirical window demonstrates 3-year overall survival rates of 87.9-90.0% and 5-year survival of 85.1%. ESD is less invasive than surgical resection, chemoradiotherapy can be safely initiated once the esophageal scar has formed, with 6% grade ≥3 nonhematologic adverse events (23). The 2-year locoregional control rate and overall survival rate were both 100% with early intervention.

Notably, a multicenter Japanese real-world study revealed immediate post-ESD chemoradiation significantly reduced regional/distant recurrence risk (HR = 0.27, 95% CI: 0.15-0.47; P < 0.001) without increasing overall recurrence (HR = 0.76, 95% CI: 0.46-1.27; P = 0.30) (5). These outcomes contrast with post-esophagectomy adjuvant therapy patterns, confirming the safety and efficacy of early chemoradiation post-ESD.

Limited data exist regarding delayed chemoradiotherapy. A retrospective cohort study found that esophagectomy at a median of 3 months post-ESD resulted in comparable 3-year survival rates to immediate esophagectomy (91.6% vs 90.9%; HR = 0.88, 95% CI: 0.24-3.21; P = 0.817) (32). However, the distinct therapeutic mechanisms of surgery versus chemoradiation preclude direct extrapolation. Given the absence of contraindications, prompt chemoradiotherapy initiation post-ESD is recommended to maximize oncological control while maintaining procedural safety.

### 5.2 Radiation dose optimization

The phase III ARTDECO trial established that dose escalation to 61.6 Gy failed to improve local control versus 50.4 Gy (HR=1.03, 95%CI 0.73-1.44; P=0.85) across histological subtypes in definitive chemoradiotherapy (49). This dose-independent efficacy pattern was corroborated by a multicenter randomized trial showing comparable survival between 60 Gy and 50 Gy cohorts (50). Current guidelines accordingly recommend 50 Gy/25 fractions or 50.4 Gy/28 fractions as the standard.

Chinese multicenter data reveal 87.9% 3-year overall survival with 50 Gy post-ESD (47), albeit with elevated grade ≥3 pneumonitis rates and other radiation-induced adverse events (50). Conversely, neoadjuvant protocols (CROSS/NEOCRTEC5010 trials) employing 41.4 Gy/23 fractions or 40 Gy/20 fractions achieved 43-48% pathological complete response rates (51–54), suggesting potential for dose de-escalation in adjuvant settings.

Regional practices reflect this paradigm shift. Japanese cohorts receiving 41.4 Gy/23 fractions demonstrate a 3-year overall survival rate exceeding 90%, with a 5-year survival rate of 85.1% (39, 48). Similarly, Chinese study with 40 Gy/20 fractions reports a 2-year overall survival rate of 100% (23).

Despite these advances, the optimal radiation dose remains uncertain. Current clinical consensus recommends 41.4 Gy/23 fractions or 40 Gy/20 fractions are recommended based on comparable survival outcomes and superior safety profiles.

### 5.3 Target volume delineation strategies

Additional chemoradiotherapy following ESD aims to mitigate local recurrence through precise radiation field design, with ongoing debate regarding two critical aspects (1): inclusion of the primary tumor bed in gross tumor volume delineation, and (2) selection between involved-field irradiation versus elective nodal irradiation.

Current evidence diverges on gross tumor volume delineation. Several studies suggest encompassing the ESD resection bed as the gross tumor volume, typically irradiated with 50Gy/25 fractions to 60Gy/30 fractions (23, 47, 55). Conversely, some studies advocate omitting gross tumor volume delineation unless positive margins exist, focusing instead on prophylactic nodal coverage at 50.4 Gy/28 fractions to 60 Gy/30 fractions (39, 48, 56).

Regarding nodal irradiation strategies, elective nodal irradiation remains predominant in clinical practice, with field design dictated by tumor location (39, 48, 56). The upper thoracic lesions typically encompass supraclavicular, upper mediastinal, and subcarinal region. The middle esophageal tumors include mediastinal and perigastric regions. The lower esophageal cancers extend to celiac nodal stations.

However, emerging evidence from advanced disease studies challenges this paradigm. Involved-field irradiation demonstrates comparable survival rates to elective nodal irradiation (57–59). Furthermore, involved-field irradiation significantly reduces radiation-induced side effects incidence, attributed to reduction in normal tissues radiation exposure (60, 61). Similarly, involved-field irradiation, targeting the primary tumor with 3–5 cm craniocaudal margins and adjacent nodes regions, maintains efficacy for T1N0M0 lesions (62, 63).

Post-ESD involved-field irradiation data remains limited. A multicenter study delineated the clinical target volume as the gross tumor volume plus a 2–5 cm craniocaudal margin, with or without elective nodal irradiation (47). Due to the small sample size (47 patients), directly comparison between involved-field irradiation and elective nodal irradiation was performed, preventing definitive conclusions.

Current consensus increasingly favors involved-field irradiation for margin negative cases given its favorable toxicity profile, reserving elective nodal irradiation for multifocal lesions or high-risk histopathological features. Radiation oncologists must balance recurrence prevention against organ preservation benefits, particularly in patients with pre-existing cardiopulmonary compromise.

### 5.4 Role of concurrent chemotherapy

For patients with stage T1N0M0 disease, concurrent chemoradiotherapy yields significantly inferior 5-year progression-free survival compared to esophagectomy (71.6% vs. 81.7%), while 5-year overall survival remains comparable between modalities (85.5% vs. 86.5%; HR = 1.05, 95% CI: 0.67-1.64) (64). Real-world evidence corroborates the result (65), supporting its role as a viable alternative for surgically ineligible T1N0M0 cases.

Contrasting data emerge from the KROG 21–10 retrospective study, where chemotherapy failed to independently predict overall survival (HR=0.16, 95% CI: 0.02-1.11, P = 0.06), despite improving 3-year locoregional control (94.4% vs. 66.8%, P = 0.001) (63). These paradoxical outcomes, compounded by retrospective design and small sample size, underscore the need for cautious interpretation while highlighting critical knowledge gaps regarding chemotherapy necessity post-ESD.

Radiotherapy monotherapy demonstrates comparable survival outcomes to chemoradiation in select cohorts. A multicenter study reported that radiotherapy improved 5-year overall survival (91.7% vs 59.5%, P = 0.050) and disease-free survival (92.9% vs 42.6%, P = 0.010) compared to observation (47). Notably, these survival benefits mirrored those reported for chemoradiotherapy in contemporary series (39, 55, 66), suggesting radiotherapy monotherapy may provide comparable oncologic outcomes with reduced chemotoxicity.

However, these studies are constrained by methodological heterogeneity. The predominantly retrospective study designs lack direct comparative arms, resulting in a grade C level of evidence according to established classification systems. This heterogeneity in research methodologies significantly limits our ability to draw definitive conclusions regarding the therapeutic value of concurrent chemotherapy in this specific clinical context.

Furthermore, pharmacological evidence demonstrates that chemotherapeutic agents such as 5-fluorouracil and cisplatin may enhance radiosensitivity through synergistic mechanisms involving DNA damage stabilization, repair pathway inhibition, and tumor cell cycle synchronization during radiation exposure (67). Nevertheless, marked discrepancies in chemotherapeutic regimens across clinical trials compromise the therapeutic impact of concurrent chemotherapy on post-ESD survival outcomes.

Pending the availability of large-scale, multicenter prospective randomized controlled trials, clinicians should exercise prudence when considering additional chemotherapy recommendations. Decision-making frameworks should incorporate multidimensional risk assessment models integrating histopathological parameters (particularly tumor invasion depth), comorbidity profiles, and metastatic potential. Development of validated prognostic algorithms containing these variables may ultimately establish evidence-based recommendations for therapeutic escalation.

## 6 Conclusion

Our review highlights critical gaps between current practice patterns and evidence-based recommendations for post-ESD management. While guidelines prioritize esophagectomy after ESD with high-risk patients, our review suggests chemoradiotherapy achieves comparable survival with superior quality of life metrics (68). This discrepancy warrants urgent guideline updates to incorporate additional chemoradiotherapy as an alternative option for select patients. Furthermore, our review suggests that multidisciplinary decision-making should be performed in clinical practice based on risk-stratified recommendations and shared decision-making tools.

Several limitations must be acknowledged. First, the majority of included studies were retrospective observational analyses, which inherently carry selection bias and confounding risks. Second, significant heterogeneity exists in radiation dosing protocols, target volume definitions, and chemoradiotherapy regimens, limiting direct comparisons across studies. Third, the follow-up duration in many studies was relatively short, particularly for assessing late complications such as radiation-induced strictures or secondary malignancies. Fourth, the absence of randomized controlled trials comparing chemoradiotherapy with surgery directly after ESD leaves critical clinical questions unresolved, particularly regarding long-term quality of life and cost-effectiveness (46).

Future research should prioritize multicenter randomized controlled trials to compare chemoradiotherapy and surgery in terms of survival, toxicity, and patient-reported outcomes. Prospective studies are needed to standardize radiation dosing (40-41.4 Gy vs. 50-50.4 Gy), optimize target volume delineation strategies (involved-field irradiation vs. elective nodal irradiation), and evaluate the role of concurrent chemotherapy. Long-term follow-up studies are essential to assess recurrence patterns, metachronous cancer risks, late treatment-related morbidity, and patient-reported outcomes. Additionally, translational research should explore molecular mechanisms underlying chemoradiotherapy resistance, which may guide personalized adjuvant therapy.

## References

[1] Sung H Ferlay J Siegel RL Laversanne M Soerjomataram I Jemal A. Global cancer statistics 2020: GLOBOCAN estimates of incidence and mortality worldwide for 36 cancers in 185 countries. CA Cancer J Clin. (2021) 71:209–49. doi: 10.3322/caac.21660 33538338

[2] Morgan E Soerjomataram I Rumgay H Coleman HG Thrift AP Vignat J. The global landscape of esophageal squamous cell carcinoma and esophageal adenocarcinoma incidence and mortality in 2020 and projections to 2040: new estimates from GLOBOCAN 2020. Gastroenterology. (2022) 163:649–58.e2. doi: 10.1053/j.gastro.2022.05.054 35671803

[3] Lagergren J Smyth E Cunningham D Lagergren P. Oesophageal cancer. Lancet. (2017) 390:2383–96. doi: 10.1016/S0140-6736(17)31462-9 28648400

[4] Yeh JH Huang RY Lee CT Lin CW Hsu MH Wu TC. Long-term outcomes of endoscopic submucosal dissection and comparison to surgery for superficial esophageal squamous cancer: a systematic review and meta-analysis. Therap Adv Gastroenterol. (2020) 13:1756284820964316. doi: 10.1177/1756284820964316 PMC7656883 33224272

[5] Katada C Yokoyama T Hirasawa D Iizuka T Kikuchi D Yano T. Curative management after endoscopic resection for esophageal squamous cell carcinoma invading muscularis mucosa or shallow submucosal layer-multicenter real-world survey in Japan. Am J Gastroenterol. (2023) 118:1175–83. doi: 10.14309/ajg.0000000000002106 36624037

[6] Phan K Tian DH Cao C Black D Yan TD. Systematic review and meta-analysis: techniques and a guide for the academic surgeon. Ann Cardiothorac Surg. (2015) 4:112–22. doi: 10.3978/j.issn.2225-319X.2015.02.04 PMC4384252 25870806

[7] Moher D Liberati A Tetzlaff J Altman DG Group P. Preferred reporting items for systematic reviews and meta-analyses: the PRISMA statement. BMJ. (2009) 339:b2535. doi: 10.1136/bmj.b2535 19622551 PMC2714657

[8] Kitagawa Y Uno T Oyama T Kato K Kato H Kawakubo H. Esophageal cancer practice guidelines 2017 edited by the Japan Esophageal Society: part 1. Esophagus. (2019) 16:1–24. doi: 10.1007/s10388-018-0641-9 30171413 PMC6510883

[9] Kitagawa Y Uno T Oyama T Kato K Kato H Kawakubo H. Esophageal cancer practice guidelines 2017 edited by the Japan esophageal society: part 2. Esophagus. (2019) 16:25–43. doi: 10.1007/s10388-018-0642-8 30171414 PMC6510875

[10] Ishihara R Matsuura N Hanaoka N Yamamoto S Akasaka T Takeuchi Y. Endoscopic imaging modalities for diagnosing invasion depth of superficial esophageal squamous cell carcinoma: a systematic review and meta-analysis. BMC Gastroenterol. (2017) 17:24. doi: 10.1186/s12876-017-0574-0 28152974 PMC5288972

[11] Mizumoto T Hiyama T Oka S Yorita N Kuroki K Kurihara M. Diagnosis of superficial esophageal squamous cell carcinoma invasion depth before endoscopic submucosal dissection. Dis Esophagus. (2018) 31(7):dox142. doi: 10.1093/dote/dox142 29267962

[12] Kim SJ Kim GH Lee MW Jeon HK Baek DH Lee BE. New magnifying endoscopic classification for superficial esophageal squamous cell carcinoma. World J Gastroenterol. (2017) 23:4416–21. doi: 10.3748/wjg.v23.i24.4416 PMC5487505 28706424

[13] Oyama T Inoue H Arima M Momma K Omori T Ishihara R. Prediction of the invasion depth of superficial squamous cell carcinoma based on microvessel morphology: magnifying endoscopic classification of the Japan Esophageal Society. Esophagus. (2017) 14:105–12. doi: 10.1007/s10388-016-0527-7 PMC5362661 28386209

[14] Ishihara R Arima M Iizuka T Oyama T Katada C Kato M. Endoscopic submucosal dissection/endoscopic mucosal resection guidelines for esophageal cancer. Dig Endosc. (2020) 32:452–93. doi: 10.1111/den.13654 32072683

[15] Sato H Inoue H Ikeda H Sato C Onimaru M Hayee B. Utility of intrapapillary capillary loops seen on magnifying narrow-band imaging in estimating invasive depth of esophageal squamous cell carcinoma. Endoscopy. (2015) 47:122–8. doi: 10.1055/s-0034-1390858 25590187

[16] Akutsu Y Uesato M Shuto K Kono T Hoshino I Horibe D. The overall prevalence of metastasis in T1 esophageal squamous cell carcinoma: a retrospective analysis of 295 patients. Ann Surg. (2013) 257:1032–8. doi: 10.1097/SLA.0b013e31827017fc 23108117

[17] Hisano O Nonoshita T Hirata H Sasaki T Watanabe H Wakiyama H. Additional radiotherapy following endoscopic submucosal dissection for T1a-MM/T1b-SM esophageal squamous cell carcinoma improves locoregional control. Radiat Oncol. (2018) 13:14. doi: 10.1186/s13014-018-0960-y 29378603 PMC5789550

[18] Takahashi K Hashimoto S Mizuno KI Kobayashi T Tominaga K Sato H. Management decision based on lymphovascular involvement leads to favorable outcomes after endoscopic treatment of esophageal squamous cell carcinoma. Endoscopy. (2018) 50:662–70. doi: 10.1055/s-0043-124433 29272907

[19] Yamashina T Ishihara R Nagai K Matsuura N Matsui F Ito T. Long-term outcome and metastatic risk after endoscopic resection of superficial esophageal squamous cell carcinoma. Am J Gastroenterol. (2013) 108:544–51. doi: 10.1038/ajg.2013.8 23399555

[20] Yoshii T Ohkawa S Tamai S Kameda Y. Clinical outcome of endoscopic mucosal resection for esophageal squamous cell cancer invading muscularis mucosa and submucosal layer. Dis Esophagus. (2013) 26:496–502. doi: 10.1111/j.1442-2050.2012.01370.x 22676622

[21] Koterazawa Y Nakamura T Oshikiri T Kanaji S Tanaka S Ishida T. A comparison of the clinical outcomes of esophagectomy and chemoradiotherapy after noncurative endoscopic submucosal dissection for esophageal squamous cell carcinoma. Surg Today. (2018) 48:783–9. doi: 10.1007/s00595-018-1650-y PMC6060875 29532261

[22] Saeki H Watanabe M Mine S Shigaki H Oya S Ishiyama A. Esophagectomy for superficial esophageal cancer after non-curative endoscopic resection. J Gastroenterol. (2015) 50:406–13. doi: 10.1007/s00535-014-0983-6 25084980

[23] Suzuki G Yamazaki H Aibe N Masui K Sasaki N Shimizu D. Endoscopic submucosal dissection followed by chemoradiotherapy for superficial esophageal cancer: choice of new approach. Radiat Oncol. (2018) 13:246. doi: 10.1186/s13014-018-1195-7 30547811 PMC6295044

[24] Hamada K Ishihara R Yamasaki Y Hanaoka N Yamamoto S Arao M. Efficacy and safety of endoscopic resection followed by chemoradiotherapy for superficial esophageal squamous cell carcinoma: A retrospective study. Clin Transl Gastroenterol. (2017) 8:e110. doi: 10.1038/ctg.2017.36 28771241 PMC5587838

[25] Mochizuki Y Saito Y Tsujikawa T Fujiyama Y Andoh A. Combination of endoscopic submucosal dissection and chemoradiation therapy for superficial esophageal squamous cell carcinoma with submucosal invasion. Exp Ther Med. (2011) 2:1065–8. doi: 10.3892/etm.2011.319 PMC3440818 22977621

[26] Suzuki G Yamazaki H Aibe N Masui K Shimizu D Kimoto T. Radiotherapy for T1N0M0 esophageal cancer: analyses of the predictive factors and the role of endoscopic submucosal dissection in the local control. Cancers (Basel). (2018) 10(8):259. doi: 10.3390/cancers10080259 30081489 PMC6115973

[27] UChinami Y Myojin M Takahashi H Harada K Shimizu S Hosokawa M. Prognostic factors in clinical T1N0M0 thoracic esophageal squamous cell carcinoma invading the muscularis mucosa or submucosa. Radiat Oncol. (2016) 11:84. doi: 10.1186/s13014-016-0660-4 27328734 PMC4915080

[28] Motoyama S Jin M Matsuhashi T Nanjo H Ishiyama K Sato Y. Outcomes of patients receiving additional esophagectomy after endoscopic resection for clinically mucosal, but pathologically submucosal, squamous cell carcinoma of the esophagus. Surg Today. (2013) 43:638–42. doi: 10.1007/s00595-012-0295-5 22899184

[29] Nihei K Minashi K Yano T Shimoda T Fukuda H Muto M. Final analysis of diagnostic endoscopic resection followed by selective chemoradiotherapy for stage I esophageal cancer: JCOG0508. Gastroenterology. (2023) 164:296–9.e2. doi: 10.1053/j.gastro.2022.10.002 36240951

[30] Ikeda A Hoshi N Yoshizaki T Fujishima Y Ishida T Morita Y. Endoscopic submucosal dissection (ESD) with additional therapy for superficial esophageal cancer with submucosal invasion. Intern Med. (2015) 54:2803–13. doi: 10.2169/internalmedicine.54.3591 26567992

[31] Kudou M Shiozaki A Fujiwara H Konishi H Shoda K Arita T. Efficacy of additional surgical resection after endoscopic submucosal dissection for superficial esophageal cancer. Anticancer Res. (2017) 37:5301–7. doi: 10.21873/anticanres.11956 28870968

[32] Wang S Huang Y Xie J Zhuge L Shao L Xiang J. Does delayed esophagectomy after endoscopic resection affect outcomes in patients with stage T1 esophageal cancer? A propensity score-based analysis. Surg Endosc. (2018) 32:1441–8. doi: 10.1007/s00464-017-5830-4 28916920

[33] Eguchi T Nakanishi Y Shimoda T Iwasaki M Igaki H Tachimori Y. Histopathological criteria for additional treatment after endoscopic mucosal resection for esophageal cancer: analysis of 464 surgically resected cases. Mod Pathol. (2006) 19:475–80. doi: 10.1038/modpathol.3800557 16444191

[34] Nozaki I Kato K Igaki H Ito Y Daiko H Yano M. Evaluation of safety profile of thoracoscopic esophagectomy for T1bN0M0 cancer using data from JCOG0502: a prospective multicenter study. Surg Endosc. (2015) 29:3519–26. doi: 10.1007/s00464-015-4102-4 PMC4648951 25676203

[35] Ancona E Rampado S Cassaro M Battaglia G Ruol A Castoro C. Prediction of lymph node status in superficial esophageal carcinoma. Ann Surg Oncol. (2008) 15:3278–88. doi: 10.1245/s10434-008-0065-1 18726651

[36] Oguma J Ozawa S Saikawa Y Kitagawa Y. Surgical treatments for squamous cell carcinoma of the esophagus reaching to the muscularis mucosa or the upper third of the submucosal layer. Oncol Lett. (2010) 1:521–5. doi: 10.3892/ol_00000092 PMC3436346 22966336

[37] Tanaka T Matono S Mori N Shirouzu K Fujita H. T1 squamous cell carcinoma of the esophagus: long-term outcomes and prognostic factors after esophagectomy. Ann Surg Oncol. (2014) 21:932–8. doi: 10.1245/s10434-013-3372-0 24232603

[38] Nozaki I Machida R Kato K Daiko H Ito Y Kojima T. Long-term survival of patients with T1bN0M0 esophageal cancer after thoracoscopic esophagectomy using data from JCOG0502: a prospective multicenter trial. Surg Endosc. (2022) 36:4275–82. doi: 10.1007/s00464-021-08768-5 34698936

[39] Minashi K Nihei K Mizusawa J Takizawa K Yano T Ezoe Y. Efficacy of endoscopic resection and selective chemoradiotherapy for stage I esophageal squamous cell carcinoma. Gastroenterology. (2019) 157:382–90.e3. doi: 10.1053/j.gastro.2019.04.017 31014996

[40] Hatta W Koike T Takahashi S Shimada T Hikichi T Toya Y. Risk of metastatic recurrence after endoscopic resection for esophageal squamous cell carcinoma invading into the muscularis mucosa or submucosa: a multicenter retrospective study. J Gastroenterol. (2021) 56:620–32. doi: 10.1007/s00535-021-01787-y 33881632

[41] Kawaguchi G Sasamoto R Abe E Ohta A Sato H Tanaka K. The effectiveness of endoscopic submucosal dissection followed by chemoradiotherapy for superficial esophageal cancer. Radiat Oncol. (2015) 10:31. doi: 10.1186/s13014-015-0337-4 25636830 PMC4316795

[42] Li JD Xu XF Yu JJ Sun LY Wang Z. Long-term outcomes of combined endoscopic resection and chemoradiotherapy for esophageal squamous cell carcinoma with submucosal invasion. Dig Liver Dis. (2018) 50:975. doi: 10.1016/j.dld.2018.03.024 29673953

[43] Tanaka T Ueno M Iizuka T Hoteya S Haruta S Udagawa H. Comparison of long-term outcomes between esophagectomy and chemoradiotherapy after endoscopic resection of submucosal esophageal squamous cell carcinoma. Dis Esophagus. (2019) 32(12):doz023. doi: 10.1093/dote/doz023 30980070

[44] Kato H Sato A Fukuda H Kagami Y Udagawa H Togo A. A phase II trial of chemoradiotherapy for stage I esophageal squamous cell carcinoma: Japan Clinical Oncology Group Study (JCOG9708). Jpn J Clin Oncol. (2009) 39:638–43. doi: 10.1093/jjco/hyp069 19549720

[45] Noordman BJ Wijnhoven BPL Lagarde SM Boonstra JJ Coene P Dekker JWT. Neoadjuvant chemoradiotherapy plus surgery versus active surveillance for oesophageal cancer: a stepped-wedge cluster randomised trial. BMC Cancer. (2018) 18:142. doi: 10.1186/s12885-018-4034-1 29409469 PMC5801846

[46] Yang Y Su Y Zhang X Liu J Zhang H Li B. Esophagectomy versus definitive chemoradiotherapy for patients with clinical stage N0 and pathological stage T1b esophageal squamous cell carcinoma after endoscopic submucosal dissection: study protocol for a multicenter randomized controlled trial (Ad-ESD Trial). Trials. (2020) 21:603. doi: 10.1186/s13063-020-04461-5 32611448 PMC7331187

[47] Yang X Zhao L Shi A Chen C Cao J Zhang Y. Radiotherapy improves survival of patients with lymphovascular invasion in pT1b esophageal squamous cell cancer after endoscopic submucosal dissection. Am J Gastroenterol. (2023) 118:1344–52. doi: 10.14309/ajg.0000000000002257 36972240

[48] Yoshimizu S Yoshio T Ishiyama A Tsuchida T Horiuchi Y Omae M. Long-term outcomes of combined endoscopic resection and chemoradiotherapy for esophageal squamous cell carcinoma with submucosal invasion. Dig Liver Dis. (2018) 50:833–8. doi: 10.1016/j.dld.2018.01.138 29477349

[49] Hulshof M Geijsen ED Rozema T Oppedijk V Buijsen J Neelis KJ. Randomized study on dose escalation in definitive chemoradiation for patients with locally advanced esophageal cancer (ARTDECO study). J Clin Oncol. (2021) 39:2816–24. doi: 10.1200/JCO.20.03697 34101496

[50] Xu Y Dong B Zhu W Li J Huang R Sun Z. A phase III multicenter randomized clinical trial of 60 Gy versus 50 Gy radiation dose in concurrent chemoradiotherapy for inoperable esophageal squamous cell carcinoma. Clin Cancer Res. (2022) 28:1792–9. doi: 10.1158/1078-0432.CCR-21-3843 35190815

[51] van Hagen P Hulshof MC van Lanschot JJ Steyerberg EW van Berge Henegouwen MI Wijnhoven BP. Preoperative chemoradiotherapy for esophageal or junctional cancer. N Engl J Med. (2012) 366:2074–84. doi: 10.1056/NEJMoa1112088 22646630

[52] Eyck BM van Lanschot JJB Hulshof M van der Wilk BJ Shapiro J van Hagen P. Ten-year outcome of neoadjuvant chemoradiotherapy plus surgery for esophageal cancer: the randomized controlled CROSS trial. J Clin Oncol. (2021) 39:1995–2004. doi: 10.1200/JCO.20.03614 33891478

[53] Yang H Liu H Chen Y Zhu C Fang W Yu Z. Long-term efficacy of neoadjuvant chemoradiotherapy plus surgery for the treatment of locally advanced esophageal squamous cell carcinoma: the NEOCRTEC5010 randomized clinical trial. JAMA Surg. (2021) 156:721–9. doi: 10.1001/jamasurg.2021.2373 PMC8223138 34160577

[54] Yang H Liu H Chen Y Zhu C Fang W Yu Z. Neoadjuvant chemoradiotherapy followed by surgery versus surgery alone for locally advanced squamous cell carcinoma of the esophagus (NEOCRTEC5010): A phase III multicenter, randomized, open-label clinical trial. J Clin Oncol. (2018) 36:2796–803. doi: 10.1200/JCO.2018.79.1483 PMC6145832 30089078

[55] Lu H Bei Y Wang C Deng X Hu Q Guo W. A retrospective cohort study to observe the efficacy and safety of Endoscopic Submucosal Dissection (ESD) with adjuvant radiotherapy for T1a-MM/T1b-SM Esophageal Squamous Cell Carcinoma (ESCC). PLoS One. (2024) 19:e0298792. doi: 10.1371/journal.pone.0298792 38386660 PMC10883569

[56] Kanie Y Okamura A Asari T Maruyama S Sakamoto K Fujiwara D. Additional treatment following noncurative endoscopic resection for esophageal squamous cell carcinoma: A comparison of outcomes between esophagectomy and chemoradiotherapy. Ann Surg Oncol. (2021) 28:8428–35. doi: 10.1245/s10434-021-10225-5 34085140

[57] Yamashita H Takenaka R Omori M Imae T Okuma K Ohtomo K. Involved-field radiotherapy (IFRT) versus elective nodal irradiation (ENI) in combination with concurrent chemotherapy for 239 esophageal cancers: a single institutional retrospective study. Radiat Oncol. (2015) 10:171. doi: 10.1186/s13014-015-0482-9 26269033 PMC4554303

[58] Lyu J Yisikandaer A Li T Zhang X Wang X Tian Z. Comparison between the effects of elective nodal irradiation and involved-field irradiation on long-term survival in thoracic esophageal squamous cell carcinoma patients: A prospective, multicenter, randomized, controlled study in China. Cancer Med. (2020) 9:7460–8. doi: 10.1002/cam4.3409 PMC7571810 32841543

[59] Liu M Zhao K Chen Y Jiang GL. Evaluation of the value of ENI in radiotherapy for cervical and upper thoracic esophageal cancer: a retrospective analysis. Radiat Oncol. (2014) 9:232. doi: 10.1186/s13014-014-0232-4 25344056 PMC4224691

[60] Chen X Zhang Y Zhou X Wang M Na F Zhou L. Involved-field irradiation or elective-nodal irradiation in neoadjuvant chemo-radiotherapy for locally-advanced esophageal cancer: comprehensive analysis for dosimetry, treatment-related complications, impact on lymphocyte, patterns of failure and survival. Front Oncol. (2023) 13:1274924. doi: 10.3389/fonc.2023.1274924 37886166 PMC10598646

[61] Dai Y Huang D Zhao W Wei J. A comparative study of elective nodal irradiation and involved field irradiation in elderly patients with advanced esophageal cancer. Front Oncol. (2023) 13:1323908. doi: 10.3389/fonc.2023.1323908 38173832 PMC10763665

[62] Nakatani Y Kato K Shoji H Iwasa S Honma Y Takashima A. Comparison of involved field radiotherapy and elective nodal irradiation in combination with concurrent chemotherapy for T1bN0M0 esophageal cancer. Int J Clin Oncol. (2020) 25:1098–104. doi: 10.1007/s10147-020-01652-7 32189155

[63] Song JY Moon SH Suh YG Kim JH Oh D Noh JM. Definitive radiotherapy in patients with clinical T1N0M0 esophageal squamous cell carcinoma: A multicenter retrospective study (KROG 21-10). Radiother Oncol. (2023) 189:109936. doi: 10.1016/j.radonc.2023.109936 37783290

[64] Kato K Ito Y Nozaki I Daiko H Kojima T Yano M. Parallel-group controlled trial of surgery versus chemoradiotherapy in patients with stage I esophageal squamous cell carcinoma. Gastroenterology. (2021) 161:1878–86.e2. doi: 10.1053/j.gastro.2021.08.007 34389340

[65] Sawada K Kotani D Yukami H Mishima S Fujiwara H Kadota T. Definitive chemoradiotherapy has comparable survival outcomes to esophagectomy in patients with clinical T1N0M0 esophageal squamous cell carcinoma: real-world data. Int J Clin Oncol. (2022) 27:1279–88. doi: 10.1007/s10147-022-02185-x 35779118

[66] Tsou YK Lee CH Le PH Chen BH. Adjuvant therapy for pT1a-m3/pT1b esophageal squamous cell carcinoma after endoscopic resection: Esophagectomy or chemoradiotherapy? A critical review. Crit Rev Oncol Hematol. (2020) 147:102883. doi: 10.1016/j.critrevonc.2020.102883 32014674

[67] Cooper JS Guo MD Herskovic A Macdonald JS Martenson JA Jr. Al-Sarraf M. Chemoradiotherapy of locally advanced esophageal cancer: long-term follow-up of a prospective randomized trial (RTOG 85-01). Radiation Therapy Oncology Group. JAMA. (1999) 281:1623–7. doi: 10.1001/jama.281.17.1623 10235156

[68] van der Wilk BJ Eyck BM Wijnhoven BPL Lagarde SM Rosman C Noordman BJ. Neoadjuvant chemoradiotherapy followed by active surveillance versus standard surgery for oesophageal cancer (SANO trial): a multicentre, stepped-wedge, cluster-randomised, non-inferiority, phase 3 trial. Lancet Oncol. (2025) 26:425–36. doi: 10.1016/S1470-2045(25)00027-0 40112851

